# Metabolic Flux Distribution during Defatting of Steatotic Human Hepatoma (HepG2) Cells

**DOI:** 10.3390/metabo6010001

**Published:** 2016-01-04

**Authors:** Gabriel Yarmush, Lucas Santos, Joshua Yarmush, Srivathsan Koundinyan, Mubasher Saleem, Nir I. Nativ, Rene S. Schloss, Martin L. Yarmush, Timothy J. Maguire, Francois Berthiaume

**Affiliations:** 1 Department of Biomedical Engineering, Rutgers University, Piscataway, NJ 08854, USA; gabriel.yarmush@gmail.com (G.Y.); lucasvcsantos2@gmail.com (L.S.); josh.yarmush@gmail.com (J.Y.); srivaths1992@gmail.com (S.K.); mubasher.saleem1@gmail.com (M.S.); nirnativ1@hotmail.com (N.I.N.); schloss@soemail.rutgers.edu (R.S.S.); yarmush@rci.rutgers.edu (M.L.Y.); 2 Center for Engineering in Medicine/Surgical Services, Massachusetts General Hospital and the Shriners Hospitals for Children, Boston, MA 02114, USA

**Keywords:** fatty liver, steatosis, defatting, beta-oxidation, mass balances, liver transplantation, hepatocytes

## Abstract

Methods that rapidly decrease fat in steatotic hepatocytes may be helpful to recover severely fatty livers for transplantation. Defatting kinetics are highly dependent upon the extracellular medium composition; however, the pathways involved are poorly understood. Steatosis was induced in human hepatoma cells (HepG2) by exposure to high levels of free fatty acids, followed by defatting using plain medium containing no fatty acids, or medium supplemented with a cocktail of defatting agents previously described before. We measured the levels of 28 extracellular metabolites and intracellular triglyceride, and fed the data into a steady-state mass balance model to estimate strictly intracellular fluxes. We found that during defatting, triglyceride content decreased, while beta-oxidation, the tricarboxylic acid cycle, and the urea cycle increased. These fluxes were augmented by defatting agents, and even more so by hyperoxic conditions. In all defatting conditions, the rate of extracellular glucose uptake/release was very small compared to the internal supply from glycogenolysis, and glycolysis remained highly active. Thus, in steatotic HepG2 cells, glycolysis and fatty acid oxidation may co-exist. Together, these pathways generate reducing equivalents that are supplied to mitochondrial oxidative phosphorylation.

## 1. Introduction

With over 15,000 patients on the waiting list for liver donations and approximately 7000 liver transplants conducted annually, 2000 patients die each year [1] of acute and chronic liver failure. Hepatic macrosteatosis, defined as abnormal lipid accumulation in hepatocytes in the form of large lipid droplets, predisposes to primary non-function following transplantation and is one of the most common causes for a donor organ removal from the donor pool [2]. Although a fatty liver can function properly within the body, it fails when it is temporarily removed from circulation due to ischemia/reperfusion (I/R) injury. Thus, pathologic analysis is conducted on all donated livers, and if more than 30% of the hepatocytes appear to be macrosteatotic, the liver is generally discarded from the donor pool. If all steatotic livers could be made suitable for transplantation, more than 1000 viable livers could be added to the donor pool annually, resulting in sufficient transplants that could halve the number of patients dying while on the waiting list [3]. Furthermore, the obesity epidemic is expected to drastically increase the proportion of steatotic livers in the donor pool; thus, it is essential that techniques to recover otherwise discarded fatty livers be developed.

Previous work using pharmacological and surgical preconditioning has shown encouraging results in the attempts to stymie ischemia-reperfusion (I/R) injury on steatotic livers. For example, pharmacological preconditioning with tacrolimus of obese Zucker rat livers marginally increased their viability after liver reperfusion, likely due to increased mitochondrial ATP levels [4,5]. Cardiotrophin-1 has been shown to have protective properties against reactive oxygen species produced during ischemia of non-steatotic pig livers [6]. Surgical ischemic preconditioning, which involves brief clamping of blood vessels followed by reperfusion, prior to removing the organ from the donor has also been shown to be effective in ameliorating I/R injury on non-steatotic human livers [7,8,9]. Nevertheless, at this point, there are no clinically approved procedures to deal with steatotic livers that exceed the commonly accepted criterion of less than 30% macrosteatosis [1].

The aforementioned approaches were designed to overcome the effects of I/R injury in fatty livers, as opposed to dealing with the excessive fat load, which is a major cause of exacerbated I/R injury in fatty livers. Recent studies suggest that the primary cause of I/R injury in steatotic livers is the abnormally high intracellular lipid content which leads to larger hepatocytes, diminished vasculature, and inferior mitochondrial functioning [10,11,12]. Furthermore, defatting steatotic hepatocytes ameliorates the effects of I/R injury *in vitro,* and defatting could be implemented in whole livers via a perfusion procedure [10,11,12,13].

To provide a rational basis for further optimization of the defatting methods, we wish to gain a better insight into the defatting processes that are rate-limiting, as well as the interaction that may exist between defatting and other liver cell functions (e.g., urea synthesis). For this purpose, we measured the metabolic fluxes in HepG2 cells that were made fatty during exposure to different defatting conditions. Fluxes were determined from measured extracellular metabolite rates of change, as well as a steady-state metabolic flux analysis (MFA) model to infer strictly intracellular fluxes. No isotopic labeling was used for our MFA, unlike other methods, such as 13C MFA [14].

## 2. Results

**Figure 1:**
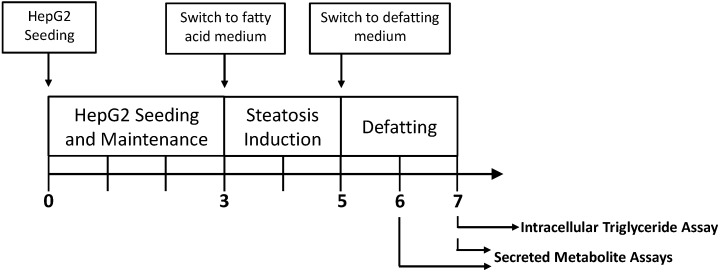
Experimental timeline for macrosteatotic induction and subsequent defatting. HepG2 cells were plated in basal medium for three days in six-well plates. Then, the HepG2 cells were exposed to the basal medium supplemented with free fatty acids to induce steatosis. Two days later, steatotic HepG2 cells were switched to defatting conditions, during which medium samples were taken daily, and cell extracts were harvested to measure intracellular triglyceride content.

HepG2 cells have been previously shown to accurately depict hepatic metabolic function [15,16,17,18,19,20], and were therefore used as a surrogate system to evaluate the effects of steatosis and subsequent defatting on liver metabolism. The cells were grown to confluence, at which point they reach a growth-arrested state, then exposed to fatty acid supplemented medium for 2 days to induce steatosis, after which different defatting conditions were tested (Figure 1). In total, four cultured conditions were studied: (A) steatotic HepG2 cells exposed to basal medium containing no fatty acids; (B) steatotic HepG2 cells exposed to basal medium containing no fatty acids but supplemented with the defatting cocktail; (C) steatotic HepG2 cells exposed to the defatting cocktail as in group (B) but, in addition, under hyperoxic conditions. The defatting cocktail is identical to that previously reported and described in the literature [10,11,12], and was previously shown to promote *in vitro* steatosis reduction by activating hepatocellular TG metabolism [21]. The fourth group (D) consisted of lean HepG2 cells that were not made steatotic by keeping them in regular basal medium throughout.

### 2.1. Triglyceride Storage

**Figure 2:**
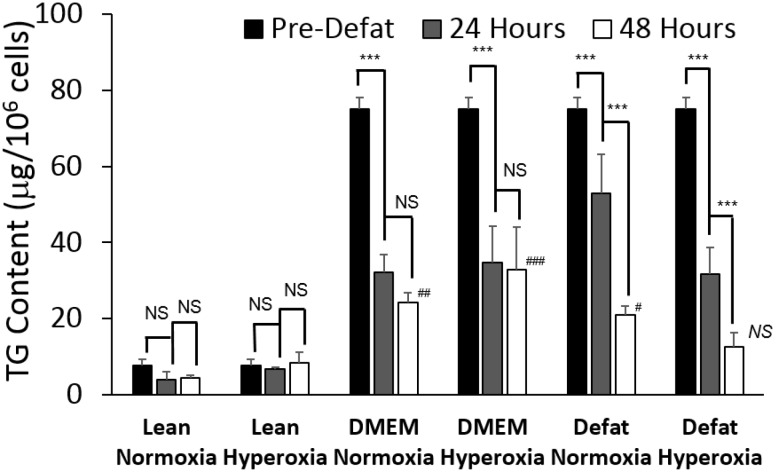
Effect of defatting condition and duration on remaining triglyceride content in steatotic HepG2s. HepG2s were made steatotic by pre-incubation with free fatty acids for two days. Then, the cells were switched to basal medium with no fatty acids (DMEM), or DMEM + defatting cocktail. In each case, defatting was performed under normoxic (21% O_2_ *v/v*) or hyperoxic (90% O_2_ *v/v*). Lean controls consisted of HepG2 cells that were never made steatotic. Data shown represent the amount of triglyceride normalized to cell number in each well, measured before defatting (pre-defat), after 24 h, and after 48 h of defatting. Values are expressed as averages ± S.E.M. for *n* = 3 replicates. ***: *p* < 0.001. NS: not significantly different. Comparisons of defatted *vs.* lean groups: ^###^ *p* < 0.001; ^##^ *p* < 0.01; ^#^ *p* < 0.05; *NS*: not significantly different.

Lean HepG2s exhibited low levels of intracellular TG, as expected. In comparison, steatotic HepG2s stored about 10 times more TG (Figure 2). When steatotic HepG2s underwent defatting in basal medium (DMEM), intracellular TG decreased by more than 50% within 24 h with no apparent effect of hyperoxia *vs.* normoxia. Furthermore, there was little to no further decrease in stored TG achieved by extending the defatting period from 24 h to 48 h under these conditions, and TG levels remained significantly higher than the lean HepG2s. In medium supplemented with defatting agents, under normoxic conditions, TG content decreased by 32% after 24 h, and by 73% after 48 h. The decrease was more pronounced under hyperoxic conditions, with a 58% decrease at 24 h, and an 83% decrease at 48 h. TG levels after hyperoxic defatting were statistically significantly lower than that observed after normoxic defatting. Furthermore, the combination of defatting agents and hyperoxia for 48 h is the only one that led to TG levels that were statistically indistinguishable from that in lean cells.

### 2.2. Measured Metabolic Fluxes

The measured metabolic fluxes are listed in Table 1, and the main observations are discussed next. Lean cells exhibited net glucose uptake and lactate release, consistent with a glycolytic metabolic pattern. Concurrently, there was a net release of glycerol and ammonia, as well as a very small release of TG and ketone bodies (beta-hydroxybutyrate and acetoacetate). Amino acid uptake rates were all positive, and together added up to a flux totaling 0.35 µg/10^6^ cells/day, which is small (about one seventh) compared to the magnitude ammonia release rates. Finally, cholesterol ester flux was negligible compared to free cholesterol, and a net uptake of cholesterol from the medium was seen.

**Table 1:** Measured metabolite rates during defatting period (µg/10^6^ cells/day) *.

Metabolite	Reaction Number	Lean Normoxia	DMEM Normoxia	Defat Normoxia	Defat Hyperoxia
**Glycolysis**					
Glucose uptake	1	9.49 ± 1.19	−1.63 ± 0.15	0.53 ± 0.18	0.70 ± 0.32
Lactate uptake	8	−14.90 ± 1.09	−1.28 ± 0.08	2.33 ± 0.33	4.80 ± 1.48
**Ketone Bodies**					
Acetoacetate release	19	−0.42 ± 0.11	0.91 ± 0.45	3.06 ± 1.11	3.88 ± 1.39
Beta−hydroxybutyrate release	20	−0.13 ± 0.04	0.75 ± 0.13	2.71 ± 0.61	3.83 ± 1.15
**Oxidative Phosphorylation**					
Oxygen uptake	21	3.79 ± 1.86	3.79 ± 1.86	3.79 ± 1.86	22.15 ± 7.16
**Amino Acid Metabolism**					
Serine (Ser) uptake	28	0.010 ± 0.001	0.006 ± 0.000	0.004 ± 0.002	0.011 ± 0.002
Glutamine (Gln) uptake	30	0.125 ± 0.040	0.106 ± 0.014	0.057 ± 0.005	0.082 ± 0.010
Histidine (His) uptake	31	0.018 ± 0.009	0.020 ± 0.009	0.023 ± 0.002	0.003 ± 0.001
Aspartic acid (Asp) uptake	32	0.013 ± 0.003	0.005 ± 0.001	0.010 ± 0.004	0.013 ± 0.002
Glutamic acid (Glu) uptake	33	0.005 ± 0.002	0.001 ± 0.000	0.007 ± 0.002	0.012 ± 0.002
Glycine (Gly) uptake	34	0.002 ± 0.000	0.008 ± 0.001	0.003 ± 0.001	0.002 ± 0.000
Ammonia (NH_3_+NH_4_^+^) uptake	36	−2.530 ± 0.091	−2.530 ± 1.081	−2.530 ± 0.987	0.540 ± 0.245
Arginine (Arg) uptake	37	0.016 ± 0.008	0.064 ± 0.010	0.049 ± 0.022	0.040 ± 0.013
Threonine (Thr) uptake	38	0.022 ± 0.001	0.016 ± 0.001	0.013 ± 0.001	0.012 ± 0.006
Alanine (Ala) uptake	39	0.007 ± 0.003	0.005 ± 0.002	0.005 ± 0.000	0.002 ± 0.001
Proline (Pro) uptake	41	0.010 ± 0.002	0.007 ± 0.003	0.010 ± 0.000	0.009 ± 0.004
Cysteine (Cys) uptake	42	0.008 ± 0.003	0.007 ± 0.003	0.014 ± 0.007	0.005 ± 0.001
Tyrosine (Tyr) uptake	43	0.014 ± 0.001	0.011 ± 0.005	0.021 ± 0.007	0.008 ± 0.000
Valine (Val) uptake	45	0.003 ± 0.001	0.023 ± 0.009	0.018 ± 0.007	0.008 ± 0.003
Ornithine (Orn) uptake	46	0.001 ± 0.000	0.007 ± 0.000	0.001 ± 0.000	0.004 ± 0.001
Lysine (Lys) uptake	47	0.028 ± 0.002	0.036 ± 0.016	0.042 ± 0.018	0.039 ± 0.001
Isoleucine (Ile) uptake	48	0.029 ± 0.014	0.018 ± 0.006	0.018 ± 0.005	0.023 ± 0.001
Leucine (Leu) uptake	49	0.022 ± 0.008	0.024 ± 0.011	0.009 ± 0.001	0.021 ± 0.005
Phenylalanine (Phe) uptake	50	0.020 ± 0.002	0.007 ± 0.002	0.009 ± 0.004	0.016 ± 0.001
**Lipid Metabolism**					
Glycerol uptake	24	−63.39 ± 2.02	30.32 ± 3.92	27.42 ± 3.71	−23.14 ± 9.40
Triglyceride (TG) uptake ^#^	27	−0.75 ± 0.22	−20.10 ± 1.52	−36.35 ± 1.96	−44.27 ± 10.58
Cholesterol ester uptake	74	0.010 ± 0.002	0.003 ± 0.002	0.005 ± 0.002	0.008 ± 0.002
Free fatty acid (FFA) uptake	84	0.000 ± 0.000	0.000 ± 0.000	0.000 ± 0.000	0.000 ± 0.000
Cholesterol uptake	90	3.24 ± 0.21	6.72 ± 2.24	3.72 ± 1.59	2.91 ± 1.40

* All metabolites were measured in the culture medium. A negative value indicates a flux in the direction opposite to that shown in Figure 6. All values report uptake rates, with the exception of the ketone bodies, which show release rates into the medium; ^#^ Represents uptake into the intracellular lipid droplet pool.

Comparing lean *vs.* steatotic HepG2 cells, both in DMEM under normoxia, reveals major differences in metabolism. Steatotic HepG2s switched from net uptake to a release in glucose, while lactate release decreased by 10 fold. Glycerol changed from net release to net uptake, concomitant with a net release of ketone bodies. Triglyceride release increased ~25 fold over the lean condition. Amino acid uptake rates added up to 0.37 µg/10^6^ cells/day, and thus were virtually unchanged from that in the lean cells.

Addition of defatting cocktail caused steatotic HepG2 cells to switch from net release of lactate and glucose to net uptake of both metabolites, although the glucose uptake rate was very small (less than one tenth) compared to that observed in lean HepG2s. Furthermore, ketone body secretion was increased ~3 fold. Glycerol uptake did not significantly change, but triglyceride release increased 1.8 fold, while cholesterol uptake decreased ~2 fold, moving towards that originally seen in lean cells. Amino acid uptake rates added up to 0.31 µg/10^6^ cells/day, corresponding to a 20% decrease from what was observed in steatotic HepG2s in DMEM alone with no defatting agents.

Hyperoxic conditions did not impact glucose uptake much, but increased lactate uptake 2 fold, and there was a further increase in ketone body production compared to normoxic conditions. Glycerol switched from net uptake to net release, and triglyceride release increased by an additional 1.2 fold. Ammonia was no longer released and a net uptake was measured. Cholesterol uptake further decreased to a level slightly below that originally seen in lean cells. Amino acid uptake rates added up to 0.31 µg/10^6^ cells/day, which is identical to what was observed in similar, albeit normoxic, conditions.

### 2.3. Calculated Fluxes

**Figure 3:**
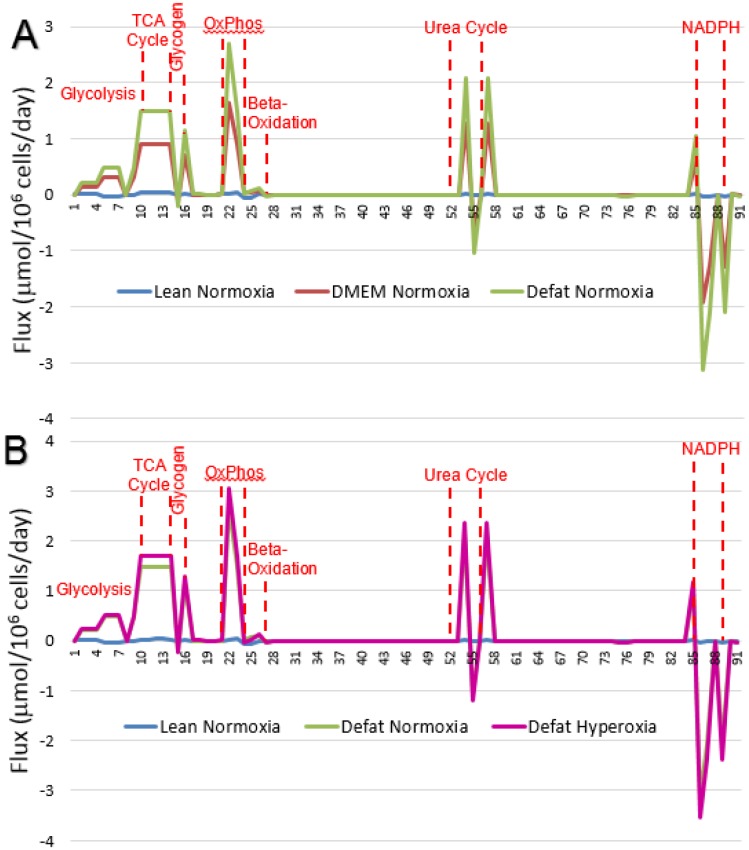
Effect of defatting condition on metabolic flux distribution. Numbers on horizontal axis correspond to reaction numbers shown in Table 2. Values shown are calculated using the MFA model with averaged measured fluxes in each group as input. (**A**) Fluxes for steatotic HepG2 cells during defatting under normoxic conditions (21% O_2_ *v/v*) in basal medium (DMEM) *vs.* medium supplemented with defatting agents (Defat). Lean controls are for cells that were never made steatotic; (**B**) Fluxes for steatotic HepG2 cells during defatting in medium supplemented with defatting agents under normoxic (21% O_2_ *v/v*) *vs.* hyperoxic (90% O_2_ *v/v*) conditions. Lean controls are for cells that were never made steatotic.

Measured fluxes were inputted into the MFA network model and calculated flux distributions are reported in detail in Table 2 and in Figure 3. Most of the changes were seen in fluxes through the pathways of glycolysis (nos. 1–9), the TCA cycle (nos. 10–14), fatty acid beta oxidation (flux no. 26), and the urea cycle (nos. 52–56).

**Table 2:** Calculated flux distribution during defatting period (µmol/10^6^ cells/day).*

Stoichiometry	Rxn Number	Lean Normoxia	DMEM Normoxia	Defat Normoxia	Defat Hyperoxia
**Glycolysis**					
Glucose + ATP --> G-6-P	1	0.010 ± 0.001	−0.002 ± 0.000	0.001 ± 0.000	0.001 ± 0.000
G-6-P --> F-6-P	2	0.013 ± 0.002	0.128 ± 0.009	0.212 ± 0.011	0.242 ± 0.059
F-6-P + ATP --> Glyceraldehyde-3-P + DHAP	3	0.013 ± 0.002	0.128 ± 0.009	0.212 ± 0.011	0.242 ± 0.059
DHAP --> Glyceraldehyde-3-P	4	0.013 ± 0.002	0.128 ± 0.009	0.212 ± 0.011	0.242 ± 0.059
Glyceraldehyde-3-P --> 3-PGA	5	−0.036 ± 0.005	0.306 ± 0.021	0.488 ± 0.025	0.505 ± 0.128
3PGA --> PEP + NADH + 2ATP	6	−0.036 ± 0.005	0.306 ± 0.021	0.488 ± 0.025	0.505 ± 0.128
PEP --> Pyruvate	7	−0.036 ± 0.005	0.306 ± 0.021	0.488 ± 0.025	0.505 ± 0.128
Pyruvate + NADH --> Lactate	8	−0.015 ± 0.001	−0.001 ± 0.000	0.002 ± 0.000	0.005 ± 0.001
Pyruvate --> Acetyl-CoA + NADH +CO2	9	−0.021 ± 0.005	0.307 ± 0.021	0.486 ± 0.025	0.500 ± 0.128
**TCA Cycle**					
Acetyl-CoA + OAA --> Citrate	10	0.033 ± 0.010	0.909 ± 0.066	1.491 ± 0.079	1.709 ± 0.414
Citrate --> alpha-KG + NADPH +CO2	11	0.033 ± 0.010	0.909 ± 0.066	1.491 ± 0.079	1.709 ± 0.414
alpha-KG --> Succinyl-CoA + NADH + CO2	12	0.034 ± 0.010	0.911 ± 0.066	1.492 ± 0.079	1.710 ± 0.414
Succinyl-CoA --> Fumarate + FADH2 + ATP	13	0.034 ± 0.010	0.911 ± 0.066	1.492 ± 0.079	1.710 ± 0.414
Fumarate --> OAA + NADH	14	0.033 ± 0.010	0.909 ± 0.066	1.491 ± 0.079	1.709 ± 0.414
**Pentose Phosphate Pathway and Glycogen Metabolism**					
(Glucose)_n-1_ + G-6-P --> (Glucose)_n_	15	−0.004 ± 0.001	−0.129 ± 0.009	−0.212 ± 0.011	−0.241 ± 0.059
G-6-P --> 12 NADPH + 6 CO2	16	0.000 ± 0.000	0.000 ± 0.000	0.000 ± 0.000	0.000 ± 0.000
Ketone Body Production					
2 Acetyl-COA --> Acetoacetyl-CoA	17	−0.002 ± 0.000	0.001 ± 0.001	0.005 ± 0.001	0.007 ± 0.002
Acetoacetyl-CoA --> Acetoacetate	18	−0.001 ± 0.000	0.002 ± 0.001	0.006 ± 0.001	0.008 ± 0.002
Acetoacetate Out	19	0.000 ± 0.000	0.001 ± 0.000	0.003 ± 0.001	0.004 ± 0.001
Acetoacetate + NADH --> B-OH butyrate	20	0.000 ± 0.000	0.001 ± 0.000	0.003 ± 0.001	0.004 ± 0.001
Oxidative Phosphorylation					
Oxygen In	21	0.004 ± 0.002	0.004 ± 0.002	0.004 ± 0.002	0.022 ± 0.007
NADH + 0.5 O2 --> 2.5 ATP	22	0.014 ± 0.019	1.641 ± 0.118	2.686 ± 0.141	3.059 ± 0.738
FADH2 + 0.5 O2 --> 2 ATP	23	0.035 ± 0.010	0.912 ± 0.066	1.493 ± 0.079	1.711 ± 0.414
Glycerol and Fatty Acid Metabolism					
Glycerol + ATP --> Glycerol-3-P	24	−0.063 ± 0.002	0.030 ± 0.004	0.027 ± 0.004	−0.023 ± 0.009
Glycerol-3-P --> Glyceraldehyde-3-P + NADH	25	−0.063 ± 0.002	0.050 ± 0.004	0.064 ± 0.004	0.021 ± 0.014
FA-CoA --> 8(9) Acetyl-CoA + 14(16) NADH	26	0.006 ± 0.001	0.067 ± 0.005	0.113 ± 0.006	0.136 ± 0.032
FA-CoA + DAG --> TG	27	−0.001 ± 0.000	−0.020 ± 0.002	−0.036 ± 0.002	−0.044 ± 0.011
**Amino Acid Metabolism**					
Ser In	28	0.000 ± 0.000	0.000 ± 0.000	0.000 ± 0.000	0.000 ± 0.000
Ser --> NH3 + Pyr	29	0.000 ± 0.000	0.000 ± 0.000	0.000 ± 0.000	0.000 ± 0.000
Gln In	30	0.000 ± 0.000	0.000 ± 0.000	0.000 ± 0.000	0.000 ± 0.000
His --> Glu + NH4+	31	0.000 ± 0.000	0.000 ± 0.000	0.000 ± 0.000	0.000 ± 0.000
Asp In	32	0.000 ± 0.000	0.000 ± 0.000	0.000 ± 0.000	0.000 ± 0.000
Glu In	33	0.000 ± 0.000	0.000 ± 0.000	0.000 ± 0.000	0.000 ± 0.000
Gly In	34	0.000 ± 0.000	0.000 ± 0.000	0.000 ± 0.000	0.000 ± 0.000
Gly --> 2 CO2 + NH3 + NADH + THF + ATP	35	0.000 ± 0.000	0.000 ± 0.000	0.000 ± 0.000	0.000 ± 0.000
NH4+ In	36	−0.003 ± 0.000	−0.003 ± 0.001	−0.003 ± 0.001	0.001 ± 0.000
Arg In	37	0.000 ± 0.000	0.000 ± 0.000	0.000 ± 0.000	0.000 ± 0.000
Thr --> Pyr + CO2 + NH4+ + 2 NADH + FADH2	38	0.000 ± 0.000	0.000 ± 0.000	0.000 ± 0.000	0.000 ± 0.000
Ala In	39	0.000 ± 0.000	0.000 ± 0.000	0.000 ± 0.000	0.000 ± 0.000
Glu + Pyr --> Ala + aKG	40	0.000 ± 0.000	0.000 ± 0.000	0.000 ± 0.000	0.000 ± 0.000
Pro In	41	0.000 ± 0.000	0.000 ± 0.000	0.000 ± 0.000	0.000 ± 0.000
Cys In	42	0.000 ± 0.000	0.000 ± 0.000	0.000 ± 0.000	0.000 ± 0.000
Tyr In	43	0.000 ± 0.000	0.000 ± 0.000	0.000 ± 0.000	0.000 ± 0.000
Tyr + aKG + 2 O2 --> Glu + CO2 + Acetoacetate + Fumarate	44	0.000 ± 0.000	0.000 ± 0.000	0.000 ± 0.000	0.000 ± 0.000
Val + aKG --> Glu + CO2 + 2NADH + FADH2 + Succ-CoA	45	0.000 ± 0.000	0.000 ± 0.000	0.000 ± 0.000	0.000 ± 0.000
Orn In	46	0.000 ± 0.000	0.000 ± 0.000	0.000 ± 0.000	0.000 ± 0.000
Lys + 2 aKG + NADPH --> 2Glu + Acetoacetyl-CoA + 2CO2 + 4 NADH + FADH2	47	0.000 ± 0.000	0.000 ± 0.000	0.000 ± 0.000	0.000 ± 0.000
Ile + aKG --> Glu + Succ-CoA + Acetyl-CoA + NADH + FADH2	48	0.000 ± 0.000	0.000 ± 0.000	0.000 ± 0.000	0.000 ± 0.000
Leu + aKG --> Glu + Acetyl-CoA + Acetoacetate + CO2 + NADH + FADH2	49	0.000 ± 0.000	0.000 ± 0.000	0.000 ± 0.000	0.000 ± 0.000
Phe + O2 --> Tyr	50	0.000 ± 0.000	0.000 ± 0.000	0.000 ± 0.000	0.000 ± 0.000
Glu + Cys + Gly --> GSH	51	0.000 ± 0.000	0.000 ± 0.000	0.000 ± 0.000	0.000 ± 0.000
**Urea Cycle**					
HCO3- + NH4+ + Orn + 2 ATP --> Citrulline	52	−0.002 ± 0.000	−0.002 ± 0.000	−0.002 ± 0.000	−0.002 ± 0.000
Citrulline + Asp + ATP --> Fumarate + Arginine	53	−0.002 ± 0.000	−0.002 ± 0.000	−0.002 ± 0.000	−0.002 ± 0.000
Arginine --> Orn + Urea	54	0.023 ± 0.014	1.276 ± 0.092	2.089 ± 0.110	2.366 ± 0.576
Urea Out	55	−0.013 ± 0.007	−0.638 ± 0.046	−1.046 ± 0.055	−1.182 ± 0.288
Orn + alpha-KG + 0.5 NADPH + 0.5 NADH --> Pro	56	0.001 ± 0.000	0.001 ± 0.000	0.001 ± 0.000	0.001 ± 0.000
**Amino Acid Metabolism (Cont’d)**					
Gln --> Glu + NH4+	57	0.015 ± 0.015	1.267 ± 0.092	2.083 ± 0.110	2.407 ± 0.576
Asp + NH4+ --> Asn	58	0.000 ± 0.000	0.000 ± 0.000	0.000 ± 0.000	0.000 ± 0.000
Thr --> Pyr + CO2 + NH4+ + 2 NADH + FADH2	59	0.000 ± 0.000	0.000 ± 0.000	0.000 ± 0.000	0.000 ± 0.000
Val + aKG --> Glu + CO2 + 2NADH + FADH2 + Succ-CoA	60	0.000 ± 0.000	0.000 ± 0.000	0.000 ± 0.000	0.000 ± 0.000
Lys + 2 aKG + NADPH --> 2Glu + Acetoacetyl-CoA + 2CO2 + 4 NADH + FADH2	61	0.000 ± 0.000	0.000 ± 0.000	0.000 ± 0.000	0.000 ± 0.000
Ile + aKG --> Glu + Succ-CoA + Acetyl-CoA + NADH + FADH2	62	0.000 ± 0.000	0.000 ± 0.000	0.000 ± 0.000	0.000 ± 0.000
Leu + aKG --> Glu + HMG-CoA + NADH + FADH2	63	0.000 ± 0.000	0.000 ± 0.000	0.000 ± 0.000	0.000 ± 0.000
Phe + O2 --> Tyr	64	0.000 ± 0.000	0.000 ± 0.000	0.000 ± 0.000	0.000 ± 0.000
**Phospholipid, Sphingolipid, and Cholesterol Metabolism**					
Ser + 1 Palm-CoA + 1 FA-CoA + NADPH --> Ceramide + CO2 + FADH2	65	0.000 ± 0.000	0.000 ± 0.000	0.000 ± 0.000	0.000 ± 0.000
Ceramide + Phosphatidylcholine --> Sphingomyelin	66	0.000 ± 0.000	0.000 ± 0.000	0.000 ± 0.000	0.000 ± 0.000
Acetoacetyl-CoA + Acetyl-CoA --> HMG-CoA	67	0.000 ± 0.000	0.000 ± 0.000	0.000 ± 0.000	0.000 ± 0.000
HMG-CoA + 2 NADPH (+ 3ATP) --> IPP	68	0.000 ± 0.000	0.000 ± 0.000	0.000 ± 0.000	0.000 ± 0.000
2 IPP --> Geranyl-PP	69	0.000 ± 0.000	0.000 ± 0.000	0.000 ± 0.000	0.000 ± 0.000
Geranyl-PP + IPP --> Farnesyl-PP	70	0.000 ± 0.000	0.000 ± 0.000	0.000 ± 0.000	0.000 ± 0.000
2 Farnesyl-PP + 0.5 NADPH + 0.5 NADH --> Squalene	71	0.000 ± 0.000	0.000 ± 0.000	0.000 ± 0.000	0.000 ± 0.000
Squalene + O2 + NADPH --> Lanosterol	72	0.000 ± 0.000	0.000 ± 0.000	0.000 ± 0.000	0.000 ± 0.000
Lanosterol + 10.5 NADPH + 4.5 NADH + 10 O2 --> Chol + 3 CO2	73	0.000 ± 0.000	0.000 ± 0.000	0.000 ± 0.000	0.000 ± 0.000
Cholesterol Ester --> Chol + FA-CoA	74	0.000 ± 0.000	0.000 ± 0.000	0.000 ± 0.000	0.000 ± 0.000
2 FA-CoA + Glycerol-3-P --> Phosphatidate	75	−0.001 ± 0.000	−0.020 ± 0.002	−0.036 ± 0.002	−0.044 ± 0.011
Phosphatidate --> DAG	76	−0.001 ± 0.000	−0.020 ± 0.002	−0.036 ± 0.002	−0.044 ± 0.011
Phosphatidate --> CDP-DAG	77	0.000 ± 0.000	0.000 ± 0.000	0.000 ± 0.000	0.000 ± 0.000
CDP-DAG + Ser --> PhosphatidylSerine	78	0.000 ± 0.000	0.000 ± 0.000	0.000 ± 0.000	0.000 ± 0.000
PhosphatidylSerine --> PhosphatidylEthanolamine + CO2	79	0.000 ± 0.000	0.000 ± 0.000	0.000 ± 0.000	0.000 ± 0.000
CDP-DAG + G-3-P --> PhosphatidylGlycerol	80	0.000 ± 0.000	0.000 ± 0.000	0.000 ± 0.000	0.000 ± 0.000
2 PG --> Cardiolipin + Glycerol	81	0.000 ± 0.000	0.000 ± 0.000	0.000 ± 0.000	0.000 ± 0.000
DAG + CDP-Choline --> PhosphatidylCholine	82	0.000 ± 0.000	0.000 ± 0.000	0.000 ± 0.000	0.000 ± 0.000
DAG + CDP-Ethanolamine --> PhosphatidylEthanolamine	83	0.000 ± 0.000	0.000 ± 0.000	0.000 ± 0.000	0.000 ± 0.000
Cholesterol Out	84	0.000 ± 0.000	0.000 ± 0.000	0.000 ± 0.000	0.000 ± 0.000
**Polyamine and NADPH**					
Arg --> Putrescine + 2 CO2 + 2 NH3	85	0.011 ± 0.007	0.637 ± 0.046	1.045 ± 0.055	1.181 ± 0.288
Arg --> Putrescine + CO2 + Urea	86	−0.036 ± 0.022	−1.914 ± 0.138	−3.134 ± 0.165	−3.548 ± 0.864
Putrescine + alpha-KG + O2 --> NH3 + NADH + Glu + NADPH + Succinate	87	−0.022 ± 0.015	−1.273 ± 0.092	−2.087 ± 0.110	−2.364 ± 0.576
Putrescine + 2 alpha-KG --> 1.5 NADH + 2 Glu + 0.5 NADPH + Succinate	88	−0.003 ± 0.000	−0.004 ± 0.002	−0.003 ± 0.002	−0.003 ± 0.001
Glu + 2 NADPH --> Pro	89	−0.025 ± 0.015	−1.278 ± 0.092	−2.090 ± 0.110	−2.367 ± 0.576
FA --> FA-CoA	90	0.003 ± 0.000	0.007 ± 0.002	0.004 ± 0.002	0.003 ± 0.001
8 Acetyl-CoA + 14 NADPH --> Palm-CoA	91	−0.001 ± 0.000	−0.020 ± 0.002	−0.036 ± 0.002	−0.044 ± 0.011

* data shown are averages of *n* = 3 experiments ± S.E.M. A negative value indicates flux is in the opposite direction of that shown in Figure 6.

As shown in Figure 3A, lean HepG2s generally exhibited low flux values for these pathways. In fatty HepG2s in basal DMEM under a normoxic atmosphere (same conditions as the lean cells), the fluxes were greater for most of the aforementioned reactions. With the fatty HepG2s, addition of the defatting cocktail to basal DMEM increased glycolysis by ~60%, TCA cycle fluxes by 64%, oxidative phosphorylation by 64%, beta oxidation by 69% and urea cycle flux by 64%. As shown in Figure 3B, switching from normoxic to hyperoxic conditions in the presence of the defatting cocktail had little to no effect on most of the glycolytic pathway (increase of ~3% in pyruvate generation), but increased the other aforementioned fluxes by about 15%.

**Figure 4:**
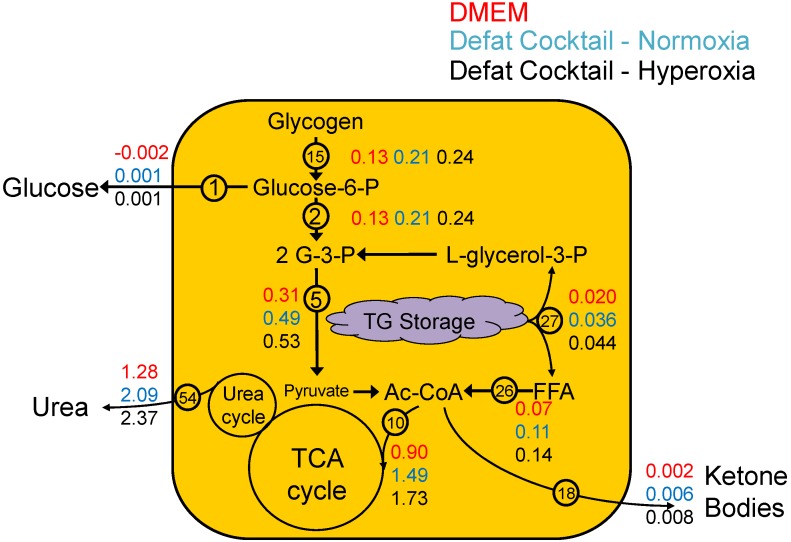
Summary of major fluxes during defatting of steatotic HepG2s. Values shown are representative fluxes (reaction number is shown) for each major pathway from Table 2 estimated by MFA during defatting under normoxic conditions with basal medium (DMEM), medium supplemented with defatting agents, and under hyperoxic conditions with medium supplemented with defatting agents.

An integrated view of the major changes is shown in Figure 4. The extracellular glucose fluxes were generally low and most of the carbon source for glycolysis was derived from glycogen. These fluxes were increased by switching to medium containing defatting cocktail, and even more by adding hyperoxic conditions. TCA cycle fluxes increased in parallel, although to a higher extent in relative terms. The acetyl-CoA fed into the TCA cycle originated from glycolysis via decarboxylation of pyruvate, and from beta-oxidation of free fatty acids (FFA) liberated from TG storage by lipolysis. Thus, the additional TCA cycle flux not accounted for by glycolytic changes could be attributed to breakdown and oxidation of stored TG. Ketone body output increased proportionally; however, the values were minimal suggesting that the bulk of the acetyl-CoA went into the TCA cycle. Urea cycle fluxes paralleled the changes in the TCA cycle, although they did not play a direct role in TG removal.

### 2.4. Urea Flux Validation

**Figure 5:**
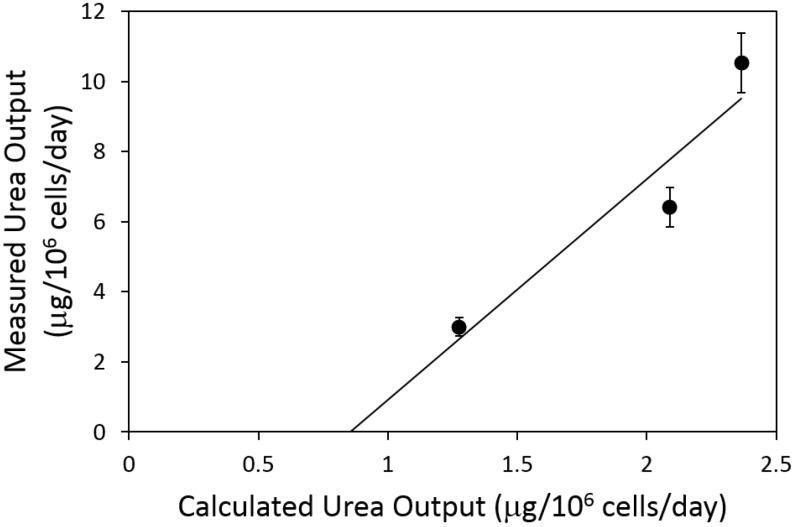
Comparison of measured and calculated urea fluxes. The measured urea secretion rate is plotted against the urea cycle output predicted by MFA using the data set shown in Table 1. Measured values are expressed as averages ± S.E.M. for *n* = 3 replicates. The least-square fit line is shown. R^2^ = 0.89.

Urea secretion was measured experimentally but not used as input to the MFA model. Thus, we compared measured *vs.* calculated values to assess the ability of the MFA model to predict fluxes. Values of measured *vs.* calculated urea fluxes for the three defatting conditions (normoxic basal DMEM, normoxic DMEM + defatting cocktail, hyperoxic DMEM+defatting cocktail) are reported in Figure 5. The data exhibited a linear correlation (R^2^ = 0.89); however, calculated fluxes tended to be approximately one third of the measured values.

## 3. Discussion

In this study, we explored the metabolic flux distribution in HepG2 cells that were made severely steatotic by exposure to high levels of free fatty acids. This *in vitro* model of hepatic steatosis (also known as fatty liver) can be used to screen for agents that decrease fat content in the liver [10,11,12]. Upon switching to regular culture medium (containing no free fatty acid supplement), lipid storage decreased over time, as previously reported in HepG2 cells as well as in adult rat hepatocytes [11,22]. Accelerated defatting was achieved by adding a cocktail of defatting agents, as well as shifting the cultures from normoxic to hyperoxic conditions. A recent study suggested that a similar defatting cocktail increased beta oxidation as the main route for lipid clearance [11]. The MFA findings herein suggest the same, whereby fluxes that generate acetyl-CoA from stored TG increased in proportion with the rate of TG removal (Figure 4). An unexpected result, however, is that the increased fatty acid oxidation occurred in parallel with an increase in glycolysis. The liver is typically glycolytic when the host is in a fed state, a condition which favors fatty acid synthesis and increased lipid storage. Conversely, fatty acid oxidation in liver takes place when the whole organism is fasting and the liver uses ATP derived from this process as a source of energy to drive glucose synthesis by gluconeogenesis from lactate and other suitable carbon sources, such as amino acids [23]. Thus, the metabolic state of severely steatotic HepG2 cells observed during defatting, which involves concurrent glycolysis and lipid oxidation, was uniquely different. One would normally associate hepatic beta-oxidation as a response to fasting, with concomitant increases in ketone body and glucose production, but this unusual metabolic state has previously been observed [11]. Furthermore, the particular combination of agents in the defatting medium does not correspond to a physiologically relevant condition; therefore, it is not surprising that an uncommon flux distribution is observed.

The MFA results suggest that the glycolytic pathway was supplied by a breakdown of glycogen, and this supply increased *vs.* control basal medium when using defatting cocktail, and even further when using hyperoxic conditions together with the defatting cocktail. The availability of glucose via glycogenolysis is likely the reason for the lack of gluconeogenesis in our system; it is possible that gluconeogenesis would be activated once glycogen is depleted. Interestingly, the majority of the glucose 6-phosphate from glycogen did not result in a substantial release of glucose into the extracellular medium (flux no. 1); rather, the substrate was used for glycolysis. Furthermore, the rate of pyruvate conversion into lactate (flux no. 8) was very small compared to the decarboxylation of pyruvate into acetyl-CoA (flux no. 9); thus glycogen was mainly oxidized in the TCA cycle.

The substantial increase in TCA cycle fluxes during defatting, using glycogen (via glucose 6-phosphate) and triglyceride (via liberated free fatty acids), is expected to lead to a vast increase in the production of reducing equivalents, and possibly ATP, depending on the efficiency of mitochondrial oxidative phosphorylation. In a prior study, it was found that ATP levels in hepatocytes were slightly higher when defatting agents and defatting agents + hyperoxia were used [10]. Free fatty acids have been reported to cause mitochondrial uncoupling, which decreases the amount of ATP generated per molecule of NAD(P)H oxidized (sometimes referred to as the P/O ratio). It is possible that the defatting conditions, by liberating fatty acids in the cell, also decrease the P/O ratio. Recent pre-clinical studies in mice have shown that mitochondrial uncouplers are effective and potentially promising therapeutic compounds to decrease fat content in the liver [24].

The calculated urea cycle flux, when compared to the measured production of urea (Figure 5), reveals a reasonably good correlation (R^2^ = 0.89), although the measured urea output was three to four times greater than the predicted urea cycle flux. Our flux calculations also predicted high values for reactions that involve putrescine (fluxes no. 85–87). However, these fluxes were expected to be smaller because putrescine and other polyamines are known to be synthesized in small amounts as these molecules mostly play regulatory roles that remain to be fully elucidated [25]. It is noteworthy that the nitrogen input via amino acids and ammonia in Table 1 minus the measured urea release adds up to a net loss of nitrogen ranging from 1 to 5 µmol nitrogen/10^6^ cells/day. A net release of nitrogen is highly suggestive of endogenous protein degradation in the cells. This potential source of nitrogen was not included in the MFA model because it is challenging to experimentally measure this parameter and it has a minor impact on central carbon metabolism [26], which is the most relevant to lipid clearance. It is unclear whether increased urea cycle fluxes play a useful role in the defatting process, or are merely a by-product of the increased activity of the TCA cycle and oxidative phosphorylation pathways.

The most complete defatting was obtained when the defatting cocktail, together with hyperoxia, were applied to the steatotic HepG2s for 48 h. The TG levels at that point were similar to the lean cells. Such kinetics are similar to that reported for adult rat hepatocytes [11,22], but significantly slower to that obtained in one study on whole steatotic livers perfused *ex vivo* [21]. In this study, we found that the rates in the mitochondrial oxidative phosphorylation reactions were increased several fold in steatotic HepG2 cells compared to the lean HepG2 cells. Furthermore, defatting rate was increased when switching to hyperoxic conditions; thus, oxygen transport to the cells in the static HepG2 culture system clearly augments defatting performance. It is therefore plausible that flow perfusion, by enhancing oxygen delivery to the cells, increases the defatting rate in perfused livers above and beyond what is observed in the HepG2 cultures.

In conclusion, using MFA, we quantitatively described the effects of defatting agents on central carbon metabolism in steatotic HepG2 cells. MFA suggests that in the absence of exogenous free fatty acids in the culture medium, steatotic HepG2 cells decrease their fat content over time. The defatting occurs primarily via beta-oxidation followed by complete oxidation of the generated acetyl-CoA in the TCA cycle. During this time, glycolysis and glycogenolysis remained highly active and also supplied acetyl-CoA to the TCA cycle. The addition of defatting agents enhanced the same fluxes, which could be further increased by the use of hyperoxic conditions. Taken together, these pathways increase the generation of reducing equivalents that are supplied to mitochondrial oxidative phosphorylation.

## 4. Materials and Methods

### 4.1. Materials

HepG2 cells were purchased from the American Type Tissue Culture Collection (ATCC; Manassas, VA, USA). Oleic acid and linoleic acid were from Sigma-Aldrich (St. Louis, MO, USA). Bovine serum albumin (BSA), purified by heat shock fractionation, was from Sigma-Aldrich (cat. number A7906). Dulbecco’s modified Eagle’s medium (DMEM; with 4.5 g/L D-glucose, L-glutamine, and 110 mg/L sodium pyruvate), penicillin-streptomycin (P/S), phosphate buffered saline (PBS, pH = 7.4), and fetal bovine serum (FBS) were from Thermo Scientific (Grand Island, NY, USA). Tissue culture plates and flasks were purchased from Becton-Dickinson (Franklin Lakes, NJ, USA).

### 4.2. HepG2 Cell Culture

The HepG2 cells were cultured in T-12.5 flasks and passaged at 70% confluence, using a basal medium consisting of DMEM supplemented with 10% FBS and 2% P/S, and in a 10% CO2/90% air atmosphere. The cells were then transferred into 6-well plates and grown to confluence for 3 days or more. During this time, culture medium was replaced daily with fresh medium (1 mL/well).

### 4.3. Steatotic Induction and Removal

Confluent HepG2 in 6-well plates were switched to fresh basal medium supplemented with free fatty acids (FFAs; 2 mM oleic acid + 2 mM linoleic acid) and BSA (4% *w/v*, to stabilize the fatty acids in solution), which induces intracellular steatosis, for 2 days. This period is considered to be the steatosis induction period (Figure 1). Cells were then exposed to defatting conditions, which consisted of fresh basal medium with no supplemental fatty acids, or the same with a mixture of the following: 0.01 mM forskolin, 0.001 mM GW7647, 0.01 mM hypericin, 0.01 mM scoparone, 0.001 mM GW501516 and amino acids, as previously described [10,11,12]. This cocktail was previously shown to promote *in vitro* steatosis reduction by activating hepatocellular TG metabolism [26]. Furthermore, each medium was tested under normoxic (21% O_2_ *v/v*) and hyperoxic (90% O_2_ *v/v*) atmospheres. This period is considered to be the defatting period.

### 4.4. Metabolite Measurements and Extracellular Flux Calculations

Fresh medium, and culture medium supernatants (harvested after 24 and 48 h of defatting time) were assayed for the concentration of the following 28 extracellular metabolites (also listed in Table 1): glucose, lactate, glycerol, amino acids, urea, free fatty acids, free cholesterol, cholesterol ester, acetoacetate, and beta-hydroxybutyrate. Amino acids were measured by high-performance liquid chromatography with a Beckman Coulter HPLC Gold 125 system as described in [27]. Free fatty acids were measured using a commercial kit (Boehringer Mannheim, Mannheim, Germany) based on a reaction catalyzed by acyl-CoA synthetase. Beta-hydroxybutyrate and acetoacetate were measured with Enzychrom assay kits from Gentaur (New York, NY, USA). Urea was measured based on the chromogenic product generated by reaction with diacetyl monoxime in a commercial kit (Sigma-Aldrich). Glucose was measured using a commercially available kit (Sigma-Aldrich), based on a reaction catalyzed by glucose oxidase. Lactate was measured using a commercial kit (Sigma-Aldrich) based on the conversion to pyruvate catalyzed by lactate oxidase. Free cholesterol and cholesterol ester were quantified based upon the reaction catalyzed by cholesterol oxidase using a commercial kit (Sigma-Aldrich). In addition, intracellular triglyceride levels were measured as the amount of glycerol liberated by lipase during an enzymatic assay (Sigma-Aldrich), as described in [10,11,12].

The change in the total amount of metabolite (concentration × total medium volume) between the fresh medium and the medium supernatant at the end of the defatting period, divided by the defatting time period (2 days) was calculated to yield the measured flux per cell well. That number was then normalized to the number of seeded cells.

### 4.5. MFA Model Establishment

**Figure 6:**
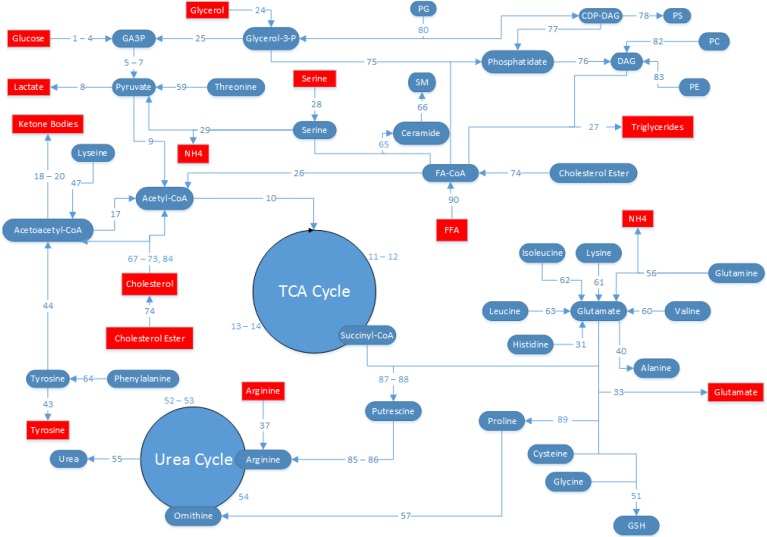
Metabolic flux analysis (MFA) reaction map. The metabolic network of the pathways considered in the MFA is shown. Blue ovals denote metabolites that are intracellular, and red rectangles denote metabolites that are extracellular. Note that triglycerides are considered to be an extracellular species because they are sequestered in lipid droplets that are physically separated from the rest of the cell. Extracellular metabolite concentrations were experimentally measured, converted into fluxes, and used as input for the MFA, with the exception of urea, which was used to compare and independently measured flux to that predicted by the MFA model.

HepG2 metabolism was represented by a network of known stoichiometric relationships among intracellular and extracellular metabolites. The pathways included in the network, as well as the numbering used for the flux equations comprising the pathways, are shown in Figure 6 and also listed in Table 1. The pathways included are summarized here (flux numbers in brackets): glycolysis (1–9), the TCA cycle (10–14), pentose-phosphate pathway (PPP) and glycogenolysis (15–16), ketogenesis (17–20), oxidative phosphorylation (21–23), FFA synthesis and oxidation (24–27, 75–76, 90), amino acid metabolism (28–51, 56, 58–64, 89), urea cycle (52–55, 57, 85–88), cholesterol synthesis (67–74, 84), and sphingolipid metabolism (65–66), phospholipid synthesis (77–83). This model was adapted from another published MFA model of fattened HepG2 cells studying free fatty acid toxicity in HepG2 cells [28,29], with the following major assumptions:

(1) The HepG2 cells are in a glycolytic state, and conversion of pyruvate into oxaloacetate by pyruvate carboxylase is assumed to be zero. All pyruvate from glycolysis is either converted to lactate by lactate dehydrogenase, or to acetyl-CoA by pyruvate dehydrogenase.
(2) Glycogen nor PPP fluxes were experimentally measured, and was therefore not possible to independently estimate each separately. Since the model predicts a net generation of glucose 6-phosphate from sources other than glucose, it was assumed that PPP fluxes were negligible (flux no.16) and the source of glucose 6-phosphate was entirely from glycogen (flux no. 15).
(3) Because we did not measure protein synthesis or degradation, we assumed that there is no contribution of protein synthesis or degradation to the amino acid fluxes. Previously, it was found that albumin (the main protein product from hepatocytes) accounted for little of central carbon and nitrogen metabolism [26].
(4) Although measurements were collected 24 h and 48 h after the onset of the defatting conditions, clearly defatting was very partial at 24 h. Therefore, we chose to calculate the average fluxes over the entire 48 h time period, and the fluxes reported here reflect a time-averaged value over 48 h.
(5) Prior reports suggest that HepG2 cells have impaired lipid secretion [30,31]; therefore, the secretion of TG into the extracellular medium was neglected compared to the flux of TG released from the intracellular droplet pool. Note that the intracellular TG, which is sequestered in lipid droplets distinct from other metabolically active compartments of the cell, was measured and handled as an extracellular metabolite as described previously [29,32,33,34,35].
(6) In the presence of high levels of FFAs, prior literature suggests that fatty acid and lipid synthesis are negligible [36,37]. Therefore, fatty acid synthesis was set to zero and all fatty acid metabolism was assumed to proceed through degradation pathways.

The system of 91 reactions is listed in Table 2. A steady-state mass balance was written around each intracellular metabolite, and the resulting system of linear equations was solved by a least-squares fit via the Moore-Penrose pseudo-inverse calculation, as previously described [28]. This method was originally described for perfused livers [38], and later modified for cultured hepatocytes [29,32,33,34,35].

### 4.6. Statistics

Experimental studies were performed in triplicate. Data are reported as average rates of uptake/release for each measured metabolite ± standard error of the mean (S.E.M.). The statistical significance of observed differences was determined using one-way ANOVA followed by Fisher’s LSD post-hoc test using KaleidaGraph (Synergy Software, Reading, PA, USA) with values of *p* < 0.05.
